# Health Tract, No. 11

**Published:** 1865

**Authors:** 


### HEALTH TRACT, No. 11.

### WALKING POSITION.

### AN ERECT POSITION ADVERSE TO CONSUMPTION.

WHO does not shrink with dread and fear at the simple mention of *Consumption?* It does not come suddenly. It begins in remote months and years agone, by imperfect breathing; by the want of frequent and *full* breaths, to keep the lungs in active operation. By this neglect, in time, the lungs swell out from a quarter to one third *less* than they ought to do; consequently, the breast flattens, the shoulders bend forward and inward, and we have the round or high shoulder, so ominous in the doctor's eye.

As consumptives *always* bend forward, and as men in high health, *candidates for aldermanic honors*, sit and walk and stand erect — *physically!* the erect position must be antagonistic to consumption, and consequently, such a position should be cultivated, sedulously cultivated, in every manner practicable; cultivated by all, not only by men, but by women and children.

No place is so well adapted to secure an *erect* locomotion as a large city; the necessity is ever present for holding up the head. Instead of giving all sorts of rules about turning out the toes, and straightening up the body, and holding the shoulders back, — all of which are impracticable to the many, because soon forgotten, or of a feeling of awkwardness and discomfort which procures a willing omission, — all that is necessary to secure the object is to *hold up the head and move on!* letting the toes and shoulders take care of themselves. Walk with the chin but slightly above a horizontal line, or with your eyes directed to things a little higher than your head. In this way you walk properly, pleasurably, and without any feeling of restraint or awkwardness.

